# Association between acetylsalicylic acid and the risk of dialysis-related infections or septicemia among incident hemodialysis patients: a nested case–control study

**DOI:** 10.1186/s12882-015-0112-7

**Published:** 2015-07-28

**Authors:** Hind Harrak, Isabelle Normand, Rachel Grinker, Naoual Elftouh, Louis-Philippe Laurin, Jean-Philippe Lafrance

**Affiliations:** Nephrology Unit, Maisonneuve-Rosemont Hospital Research Center, 5415, boul. de l’Assomption, Montreal (Quebec), H1T 2M4 Canada; Department of Medicine, University of Montreal, Montreal, Canada; Division of Nephrology, Maisonneuve-Rosemont Hospital, Montreal, Canada

**Keywords:** Epidemiology, Infections, Kidney failure, Chronic, Registries, Renal dialysis

## Abstract

**Background:**

Vascular access-related infections and septicemia are the main causes of infections among hemodialysis patients, the majority of them caused by Staphylococcus species. Acetylsalicylic acid (ASA) has recently been reported with a probable antistaphylococcal activity. This study aimed to evaluate the effect of ASA on the risk of dialysis-related infection and septicemia among incident chronic hemodialysis patients.

**Methods:**

In a nested case–control study, we identified 449 cases of vascular access-related infections and septicemia, and 4156 controls between 2001 and 2007 from our incident chronic hemodialysis patients’ cohort. Cases were defined as patients hospitalized with a main diagnosis of vascular access-related infection or septicemia on the discharge sheet (ICD-9 codes). Up to ten controls per case were selected by incidence density sampling and matched to cases on age, sex and follow-up time. ASA exposure was measured at the admission and categorized as: no use, low dose (80–324 mg/d), high dose (≥325 mg/d). Odds ratios (OR) for infections were estimated using multivariable conditional logistic regression analysis, adjusting for potential confounders.

**Results:**

Compared to no use, neither dose of ASA was associated with a decreased risk of infection: low dose (OR 1.03, 95 % CI 0.82-1.28) and high dose (OR 1.30, 95 % CI 0.96-1.75). However, diabetes (OR = 1.32, 95 % CI = 1.07–1.62) and anticoagulant use (OR = 1.62, 95 % CI = 1.30–2.02) were associated with a higher risk.

**Conclusion:**

Among hemodialysis patients, ASA use was not associated with a reduced risk of hospitalizations for dialysis-related infections or septicemia. However, ASA may remain beneficial for its cardiovascular indications.

## Background

Infections represent a great challenge to the medical field everywhere; especially among patients with comorbidities. Morbidity and mortality of infection are greater in the dialysis population compared to the general population [1–4]. In this population, infections explain more than 20 % of hospitalizations and are the second leading cause of death [5, 4, 6]. Infection-related hospitalizations (IRH) in the hemodialysis population are often associated with hemodialysis catheters, and these have been acknowledged as a major risk factor for bacteremia [7, 8]. It was estimated that more than 7000 serious complications of catheter-related bacteremia such as sepsis or metastatic infections occur annually in the United States, representing not only a burden on nephrology units, but on the cost of healthcare as well [9]. Although classic infection prevention strategies such as aseptic protocol, water quality assessment and avoidance of catheters remain essential, we are in dire of new modifiable risk factors.

While mostly used for its antiplatelet activity, acetylsalicylic acid (ASA) has been reported in vitro and in vivo as having an antistaphylococcal activity through its major metabolite, salicylic acid, mitigating α-hemolysin secretion and fibronectin binding [10]. In a relatively small study, ASA has been associated with a decreased risk of *Staphylococcus aureus* bacteremia in hemodialysis patients [11]. However, preventing one germ may not impact on the overall infection risk as other germs compete at the infection site. A survey of access-related bacteremias in hemodialysis conducted in the province of Quebec showed that *S. aureus* accounts for 54 % of isolated germs, followed by *Enterobateria* and coagulase negative *Staphylococci* with 11 % each [12]. Moreover, because the dialysis population is at high risk of bleeding events and that ASA may be potentially harmful, the potential benefit of ASA on infections should be clearly established before this new indication justifies its use [13, 14]. Therefore, this study sought to evaluate the association between ASA and the risk of dialysis-related infections or septicemia in an incident chronic hemodialysis cohort.

## Methods

### Study population and data sources

We conducted a population-based retrospective nested case–control study to assess the association between ASA and dialysis-related infections or septicemia leading to a hospitalization among incident chronic hemodialysis patients. The nested case–control design is increasingly used in epidemiologic studies. A case–control methodology is applied within a predefined cohort. The main advantage is measurement of the drug exposure at the event instead of the beginning of the follow-up, an important issue in pharmacoepidemiology since patients may start and stop medication during follow-up. A limit of this method is a slightly decreased statistical power [15, 16]. Study data were obtained from the Canadian Organ Replacement Register (CORR) and the *Régie de l’assurance maladie du Québec* (RAMQ). The CORR gathers information on organ transplantation and donation in addition to information concerning end-stage renal disease (ESRD) with respect to patients and facilities in Canada. The RAMQ is the single-payer of a provincial health insurance plan provided to all residents of the Province of Québec, Canada, that covers medical and hospital services. This administrative database provides information on all medical visits, diagnostic codes (using *International Classification of Diseases* – ICD), medical procedures during in- and outpatient encounters, and hospital discharge summaries (Med-Echo). The Med-Echo database provides details on the date of admission and discharge, primary and secondary diagnoses, and the procedures performed during the hospital stay. Moreover, all individuals aged 65 years and older, individuals on welfare and workers not insured by a private insurance company are covered by the provincial drug plan. This allowed us to obtain all drug dispensed during the study period, including date of prescription, days of supply and daily dose of more than 87 % of ESRD patients.

### Study cohort

Derivation of the incident hemodialysis cohort was described previously [17]. Patients initiating chronic hemodialysis between January 1st, 2001 and December 31st, 2007 and identified in both CORR and RAMQ data sources were included in the cohort. We excluded patients who had a prior kidney transplant or had less than 90 days of dialysis following the initiation of dialysis. This last exclusion criterion was used to ensure that our cohort included only chronic hemodialysis patients, and to make it comparable to other ESRD cohorts. To ascertain inclusion of incident patients only, we used a 2-year look back period before the first dialysis code after January 2001. Patients were followed from initiation of hemodialysis to death, kidney transplantation, end of the study period, or first hospitalization for dialysis-related infection or septicemia.

### Case definition: dialysis-related infections and septicemia

We identified all hospital admissions during the study period with an ICD-9 code (or ICD-10 after 2006) indicating a dialysis-related infection or septicemia as main diagnosis on the hospital’s discharge sheet (Table 1). If a patient was hospitalized more than once for this reason, we only considered the first hospitalization that occurred between the initiation of treatment and the end of the study period. The analysis was restricted to cases who were enrolled in the RAMQ drug plan for at least 3 months prior to the index date in order to exclude patients who might benefit from a private insurance (in which case ASA use would not be captured). The index date was considered to be the date of the first admission for cases.

**Table 1:** Definition of dialysis-related infection or septicemia (ICD-9 and ICD-10 codes and their description)

ICD-9 codes	ICD-10 codes	Description
038	A40 -A41	Streptococcal septicemia, Staphylococcal septicemia, Pneumococcal septicemia [Streptococcus pneumoniae septicemia], Septicemia due to anaerobes, Septicemia due to other gram-negative organisms, Other specified septicemias, Unspecified septicemia
112.5	B37.7	Candidal sepsis
790.7		Bacteremia
785.52	R57.2	Severe sepsis with septic shock
996.62	T82.7	Infection and inflammatory reaction due to other cardiac and vascular devices, implants and grafts, initial encounter
996.69	T85.7	Infection and inflammatory reaction due to other internal prosthetic devices, implants and grafts, initial encounter

ICD International Classification of Diseases

### Control selection

For each case, up to ten controls were randomly selected using the incidence density sampling method and matched on age (±5 years), sex, and follow-up time (±90 days). As for cases, controls had to be enrolled in the RAMQ drug plan for at least three months prior to the index date. Patients included in the cohort could serve as a case, once or several times as a control prior to being assessed as a case; or both, at different time points (explaining higher counts in controls than in the overall number of patients).

### ASA exposure

Exposure to ASA was assessed at the index date. For each ASA prescription dispensed during follow-up, we defined a prescription period, which was the time from the drug dispensation date plus the number of days supplied plus a tolerance period of 30 days [18]. For each case and control, we determined if the index date was covered by these prescription periods, and then assigned them to one of three categories: 1) not exposed; 2) a daily dose of 80 to 324 mg of ASA; or 3) a daily dose of at least 325 mg of ASA. These dose categories were selected according to prior studies showing that the antistaphyloccocal effect of ASA is independent of the antiplatelet effect and was mostly reported with a daily dose of 325 mg [11, 10, 19].

### Covariates

Age, sex, race, body mass index (BMI), smoking status, and laboratory data were measured at dialysis initiation. Estimated glomerular filtration rate (eGFR) was calculated using the four-variable MDRD equation [20]. Various co-morbidities were assessed using ICD-9 codes from the RAMQ billing database and CORR data within two years prior to dialysis initiation: cardiovascular disease, cerebrovascular disease, chronic pulmonary disease, chronic liver disease, congestive heart failure, diabetes, hyperlipidemia, hypertension, malignancy, and peripheral vascular disease. Anticoagulant and antiplatelet (other than aspirin) use was assessed at dialysis initiation using the same methodology as for ASA exposure.

### Statistical analysis

Age, BMI and laboratory values are expressed as mean and standard deviation (SD), wherever appropriate. Baseline characteristics between cases and controls were compared using the Student’s *t*-test or the Chi-squared test. Odds ratios (OR) and 95 % confidence interval (CI) were estimated using multivariable conditional logistic regression analysis. For missing laboratory values, a multiple imputation method was used [21]. In addition, an interaction analysis was performed between exposure to ASA and vascular disease, diabetes and anticoagulant use.

### Ethical considerations

This study was approved by the Government of Quebec ethics’ committee (*Commission d’accès à l’information* – CAI) and the Maisonneuve-Rosemont Hospital ethics’ committee. The ethics committee waived the requirement for informed consent for this study.

## Results and discussion

We identified a cohort of 4933 patients initiating chronic hemodialysis during the study period. 446 patients had at least one hospitalization for a dialysis-related infection or a septicemia (cases). Cases were matched to 4126 controls during the study period. The overall median follow-up time was 1.81 years (interquartile range: 0.88-3.10 years). Cases had a median time to first hospitalization of 0.92 years (interquartile range: 0.36-1.95 years). Characteristics for cases and controls are shown in Table 2. Cases were younger (66.5 *versus* 68.1 years), and had a higher prevalence of diabetes (57.9 % *versus* 53.1 %) than controls. In addition, a higher proportion of cases was using anticoagulant drugs than controls (28.9 % *versus* 21.2 %). There was no difference with respect to exposure to ASA between cases and controls.

**Table 2:** Characteristics for cases and controls

Patients’ characteristics	Cases	Controls	P-value
	*N* = 446	*N* = 4126	
Age (years)	66.5 ± 12.8	68.1 ± 10.8	0.01
Female sex (%)	42.4	40.8	0.52
Race (%)			
Black	6.0	4.1	0.16
Caucasian	84.5	86.1	
Other	9.4	9.8	
BMI (kg/m^2^)	27.8 ± 6.3	27.4 ± 6.3	0.29
Smoking (%)	15.5	14.1	0.41
Co-morbidities (%)			
Cardiovascular disease	57.9	58.2	0.87
Cerebrovascular disease	17.7	16.3	0.46
Chronic pulmonary disease	32.5	30.0	0.27
Chronic liver disease	2.2	3.1	0.33
Congestive heart failure	37.4	37.8	0.89
Diabetes	57.9	53.1	0.05
Hyperlipidemia	58.3	58.7	0.87
Hypertension	94.8	94.1	0.52
Malignancy	17.7	16.7	0.60
Peripheral vascular disease	37.4	36.7	0.75
ASA daily dose			
Unexposed	50.7	49.3	0.79
80-324 mg	35.7	37.3	
≥ 325 mg	13.7	13.4	
Other medication use (%)			
Anticoagulant	28.9	21.2	>0.001
Antiplatelet	11.0	11.8	0.63
Laboratory data			
Albumin (g/L)	33.2 ± 6.6	33.9 ± 6.5	0.09
eGFR (ml/min•1.73 m^2^)	9.0 ± 3.7	9.1 ± 4.0	0.65
Hemoglobin (g/L)	10.2 ± 1.8	10.5 ± 1.8	0.01

Units conversion: Albumin, divide by 10 to convert g/L to g/dL; Hemoglobin, divide by 10 to convert g/L to g/dL

*BMI* body mass index; *eGFR* estimated glomerular filtration rate

Adjusted OR for dialysis-related infections and septicemia are presented in Table 3. Regardless of the dose, ASA use was not associated with a reduced risk of dialysis-related infections or septicemia (OR = 1.02 [95 % CI: 0.82-1.26] for <325 mg and OR = 1.17 [95 % CI: 0.87-1.58] for ≥325 mg) compared to unexposed. Among covariates, diabetes (OR = 1.30 [95 % 1.05-1.62]) and concurrent anticoagulant use (OR = 1.61 [95 % CI: 1.29-2.01]) were associated with a higher risk of dialysis-related infection and septicemia.

**Table 3:** Adjusted odds ratios for dialysis-related infection or septicemia

Variable	Crude OR				Adjusted OR^a^			
	OR	95 % CI			OR	95 % CI		
Age (by 1 year)	0.87	0.62	,	1.23	0.86	0.71	,	1.04
Race								
Black	1.35	0.88	,	2.08	1.37	0.9	,	2.08
Caucasian	1.00	Reference			1.00	Reference		
Other	0.94	0.67	,	1.32	0.95	0.7	,	1.3
BMI (by 5 kg/m^2^)	1.05	0.97	,	1.14	1.01	0.93	,	1.11
Smoking	1.12	0.85	,	1.47	1.1	0.84	,	1.44
Co-morbidities								
Cardiovascular disease	1.11	0.90	,	1.36	1.01	0.79	,	1.28
Cerebrovascular disease	1.16	0.89	,	1.50	1.1	0.84	,	1.43
Chronic pulmonary disease	1.18	0.96	,	1.46	1.15	0.92	,	1.43
Chronic liver disease	0.77	0.40	,	1.47	0.71	0.38	,	1.32
Congestive heart failure	1.05	0.86	,	1.29	0.91	0.73	,	1.14
Diabetes	1.31	1.07	,	1.61	1.3	1.05	,	1.62
Hyperlipidemia	1.07	0.87	,	1.31	0.98	0.78	,	1.22
Hypertension	1.22	0.78	,	1.90	1.17	0.77	,	1.77
Malignancy	1.13	0.87	,	1.46	1.17	0.91	,	1.51
Peripheral vascular disease	1.09	0.88	,	1.34	1.01	0.81	,	1.24
ASA daily dose								
Unexposed	1.00	Reference			1.00	Reference		
80-324 mg	0.99	0.80	,	1.24	1.02	0.82	,	1.26
≥ 325 mg	1.06	0.78	,	1.44	1.17	0.87	,	1.58
Other medication use								
Anticoagulant	1.57	1.26	,	1.96	1.61	1.29	,	2.01
Antiplatelet	0.96	0.70	,	1.32	0.97	0.7	,	1.33
Laboratory data								
Albumin (by 10 g/L)	0.85	0.71	,	1.02	0.93	0.79	,	1.10
eGFR (ml/min•1.73 m^2^)	1.00	0.97	,	1.03	1.00	0.97	,	1.03
Hemoglobin (by 10 g/L)	0.93	0.88	,	0.99	0.94	0.89	,	1.00

Unit conversion: Albumin, multiply by 10 to convert g/dL to g/L; Hemoglobin, multiply by 10 to convert g/dL to g/L

*BMI* body mass index; *eGFR* estimated glomerular filtration rate

^a^Adjusted for demographics, body mass index, smoking, comorbidities, anticoagulant use, antiplatelet use, and laboratory values

The interaction between exposure to ASA and vascular disease (*p* = 0.72 and *p* = 0.20), diabetes (*p* = 0.59 and *p* = 0.22) or concurrent anticoagulant (*p* = 0.97 and *p* = 0.41) use was not statistically significant for low dose and high dose of ASA respectively.

The results of this large multicenter population-based study showed no association between ASA use and dialysis-related infection and septicemia in the chronic hemodialysis population.

Salicylic acid, a major metabolite of ASA, has been shown to reduce the expression of two virulence factors of *S. aureus*: α-hemolysin and fibronectin gene promoters [10]. Few observational studies evaluated the potential antimicrobial effect of ASA use as an adjunctive treatment in infective endocarditis, and found opposing results [22–24]. However, a randomized-control trial in 115 patients showed no beneficial effect and a potential increase in bleeding risk [25, 19]. To our knowledge, only one prior clinical study evaluated the potential benefit of ASA as an antimicrobial in dialysis patients. Among 872 hemodialysis patients with tunneled catheters, ASA was associated with a 54 % decreased risk of developing a *S. aureus* bacteremia [11]. These different results from our study may be explained by the fact that we considered all bacteria (and not only *S. aureus*). Indeed, when all pathogens were considered, bacteremia was not significantly reduced by ASA in the Sedlacek et al. study [11]. While reducing *S. aureus* bacteremia is important as it is associated with high morbidity and mortality, it remains important to evaluate infections from all other pathogens as they may take the place left by *S. aureus* reduction. Such a phenomenon was shown in some studies evaluating *S. aureus* nasal carriage eradication, where incidence of infections from other germs than S. aureus increased [26]. Unfortunately, microorganisms information was not available to us while conducting this study.

The dose of ASA may play an important role as earlier studies have reported an antistaphylococcal effect of ASA when using a daily dose of 325 mg, but not with the 80 mg daily dose [11, 10, 19]. This is probably explained by the fact that the antistaphylococcal effect is mediated by salicylic acid, and is therefore independent of the antiplatelet effect [10]. However, in the present study, both dose categories were not associated with decreased risk of dialysis-related infections and septicemia.

Consistent with literature, we found that diabetes was associated with an increased risk of infection [11, 8]. We also found that anticoagulant use was associated with a 61 % increase risk of dialysis-related infection and septicemia. While anticoagulant use is not a known risk factor for infection, it may be a proxy for catheter use in our cohort (anticoagulant may be used for dysfunctional catheters), which is a strong risk factor for infection [8].

Strengths of this study are its large size leading to high statistical power, the large number of included covariates, and the fact that it is conducted in a universal health care system setting, limiting potential selection bias. However, this study has also some limitations. First, because infections are identified through hospital discharge sheets, our study is limited to cases of serious dialysis-related infections and septicemia requiring hospitalizations.

The use of diagnostic codes does not prevent from underestimating the number of cases. Only the code for the main diagnosis was considered for this study. Infections recorded as secondary diagnoses could have been caused by many reasons, and their inclusion in the study would have introduced an important confounding bias limiting the interpretation of the results. While it is possible that ASA may only reduce less severe infections, we believe that we identified the most clinically important events. Because ASA can be obtained over-the-counter, our study is subject to misclassification bias. However, due to financial incentives and the facts that hemodialysis patients have already numerous medications, it is estimated that the proportion of patients using ASA chronically over-the-counter is low. Despite adjusting for various variables, our study remains prone to residual confounding. Indeed, catheter-related infections are a major cause of infection in this population, but data on catheter use was missing in our database. For reference, 54.4 % of prevalent hemodialysis patients were using a tunneled catheter in 2013 in Quebec [12]. Because the type of vascular access is not an indication or a contraindication to ASA use, we believe that its use should not differ by vascular access type.

## Conclusion

We have shown that the use of ASA is not associated with a reduced overall risk of hospitalization for dialysis-related infections or septicemia in the dialysis population. Further studies are needed to determine if ASA modifies the microorganisms’ distribution of these infections, and possibly improving outcomes. Before these studies are conducted, the potential risk of bleeding associated with ASA may limit the use of ASA for prevention of infection. However, ASA may remain beneficial for its cardiovascular indications.

## Abbreviations

ASA: Acetylsalicylic acid
BMI: Body mass index
CAI: Commission d’accès à l’information
CI: Confidence interval
CORR: Canadian Organ Replacement Register; eGFR:Estimated glomerular filtration rate
ESRD: End-stage renal disease
ICD: International classification of disease
IRH: Infection-related hospitalizations
OR: Odds ratios
RAMQ: Régie de l’assurance maladie du Québec
SD: Standard deviation
